# Accidental poisoning with aconite overdose: A case report and resuscitative emergency management in a tertiary level hospital of Bangladesh

**DOI:** 10.1002/ccr3.7845

**Published:** 2023-08-24

**Authors:** Mahabubul Islam Majumder, Ashrafur Rahaman Mahadi, Obayed Ur Rahman, Biplob Kumar Roy, Hossain Mohammad Shihab

**Affiliations:** ^1^ Department of Medicine Central Medical College Cumilla Bangladesh; ^2^ Central Medical College Cumilla Bangladesh; ^3^ Department of Anesthesiology Cumilla Medical College Cumilla Bangladesh

**Keywords:** accidental poisoning, aconite poisoning, Bangladesh, herbal medicine

## Abstract

Intoxication with aconite, a common over‐the‐counter herbal medicine in Asia, can result in ventricular tachycardia and cardiac arrest and requires heart rate monitoring in a critical care setting and aggressive use of antiarrhythmica. Educational efforts in the appropriate use of alternative medicine may help prevent intoxication.

## Graphical abstract

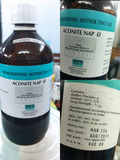

## 1 BACKGROUND

Herbal or traditional medicines have become important components of complementary medicine. Despite the prevalent perception that these medications are used to avoid the adverse effects of allopathic therapy, there is a lack of evidence about their safety. There has been an increase in adverse event reporting as the use of herbal medications has increased.^1^ This is may be due to insufficient oversight over the production and distribution of herbal goods. Furthermore, patients commonly self‐medicate with potentially dangerous items. Aconitum (*Ranunculaceae*) has hundreds of plant species that contain alkaloids including aconite, which have been associated to gastrointestinal, cardiovascular, and nervous system damage.^2^

Aconitum plants have long been utilized to cure a variety of medical conditions in China and other Asian nations.^3^ Aconite poisoning is prevalent in Asia because aconite roots (the roots and root tubers of Aconitum species) are still employed in traditional medicine for their analgesic, anti‐inflammatory, and cardiotonic properties.^4^ Despite this, aconite poisoning is common in Asia, owing to the growing popularity of herbal remedies and their ease of access.^5^

Only a few case reports and published literature have documented aconite poisoning. According to those literature, aconite acts on the voltage‐sensitive sodium channels of excitable tissues' cell membranes, such as the heart, neurons, and muscles.^3^ Patients with aconite poisoning frequently exhibit a variety of cardiovascular, neurological, gastrointestinal, and other symptoms.^6^ Neurological symptoms may be sensory (paresthesia and numbness of the face, perioral region, and four limbs) or motor (muscle weakness in the four limbs). Hypotension, chest discomfort, palpitations, bradycardia, sinus tachycardia, ventricular ectopics, ventricular tachycardia, and ventricular fibrillation are all examples of cardiovascular manifestations. Refractory ventricular tachyarrhythmias and asystole are the leading causes of mortality.^6^ The amount of Aconitum alkaloids taken is the single most important factor deciding whether severe poisoning and cardiotoxicity occur. The lethal dose is expected to be 2 mg of aconite, 5 mL of aconite tincture (herbal medicinal wine), and 1 g of raw aconite plant.^7^ Aconite tincture, according to epidemiological research, is significantly more harmful than branded medications, processed aconite root decoctions, and other aconite formulations. With aconite tincture, there is a significant danger of accidental overdose and toxicity.^4^

In this case report, we addressed the clinical management of a 35‐year‐old man who accidentally ingested 40 mL of aconite, resulting in deadly aconite poisoning. This case was presented at the Central Medical College of Cumilla, Bangladesh. This is the first case management in Bangladesh to the best of our knowledge of the relevant literature.

## 2 CASE PRESENTATION

A 34‐year‐old man presented to the emergency department, Central medical college, Bangladesh, at 3.45 a.m. with the complaints of numbness all over his body, weakness and shortness of breath, and drowsiness.

**FIGURE 1:**
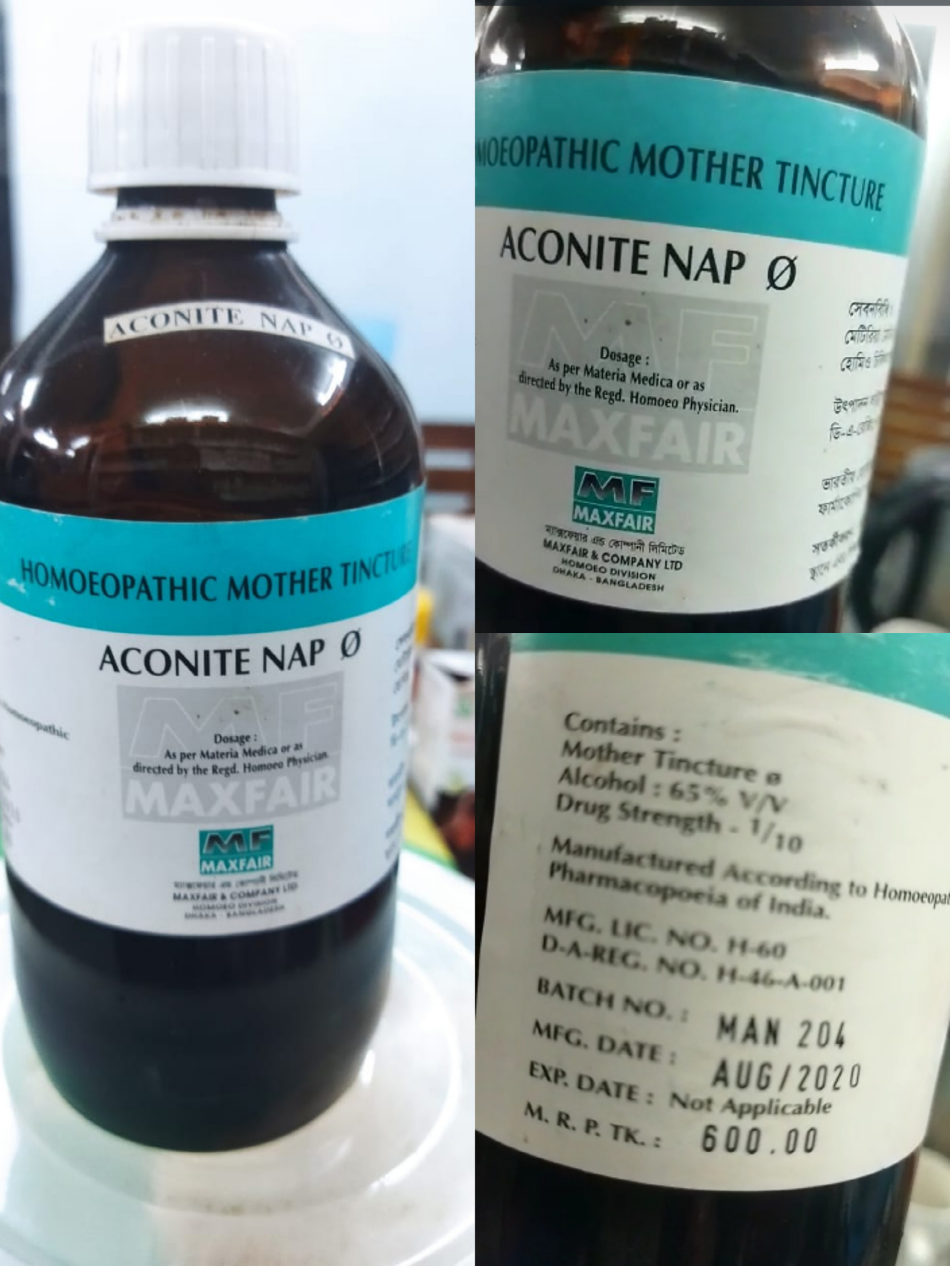
Aconitum bottle provided by the relatives.

His family members provided a history of using herbal remedies. Relatives reported the patient's accidental ingestion (40 mL) of it, which was later recognized as an Aconite (Figure 1) preparation that the patient had been taking as a topical painkiller and general well‐being. He obtained the remedy from a nearby homeopathic shop. He had these symptoms within an hour of utilizing it and came to the hospital right afterward.

His vital signs at the time of presentation were as follows: blood pressure 70/40 mmHg, heart rate 174 bpm, respiratory rate 28/min, temperature 36.3°C, and oxygen saturation 60% without oxygen with ECG showing ventricular tachyarrhythmias. He was drowsy yet arousable to painful and vocal stimuli, but he was not aware of who or where he was. The patient's respiratory sounds were equal bilaterally, with cold extremities, and there were also lateralizing signs neurologically. Upon examination, the patient's Glasgow Coma Scale score was shown to be 12, along with worsening respiratory failure and hemodynamic instability.

Supportive treatment was started right away, an 18‐gauge cannula was placed, and fluid was administered (1000 mL of normal saline). He was given high flow oxygen (15 L/min) through a non‐rebreathing mask and intravenous fluid with N/S at full rate.

**FIGURE 2:**
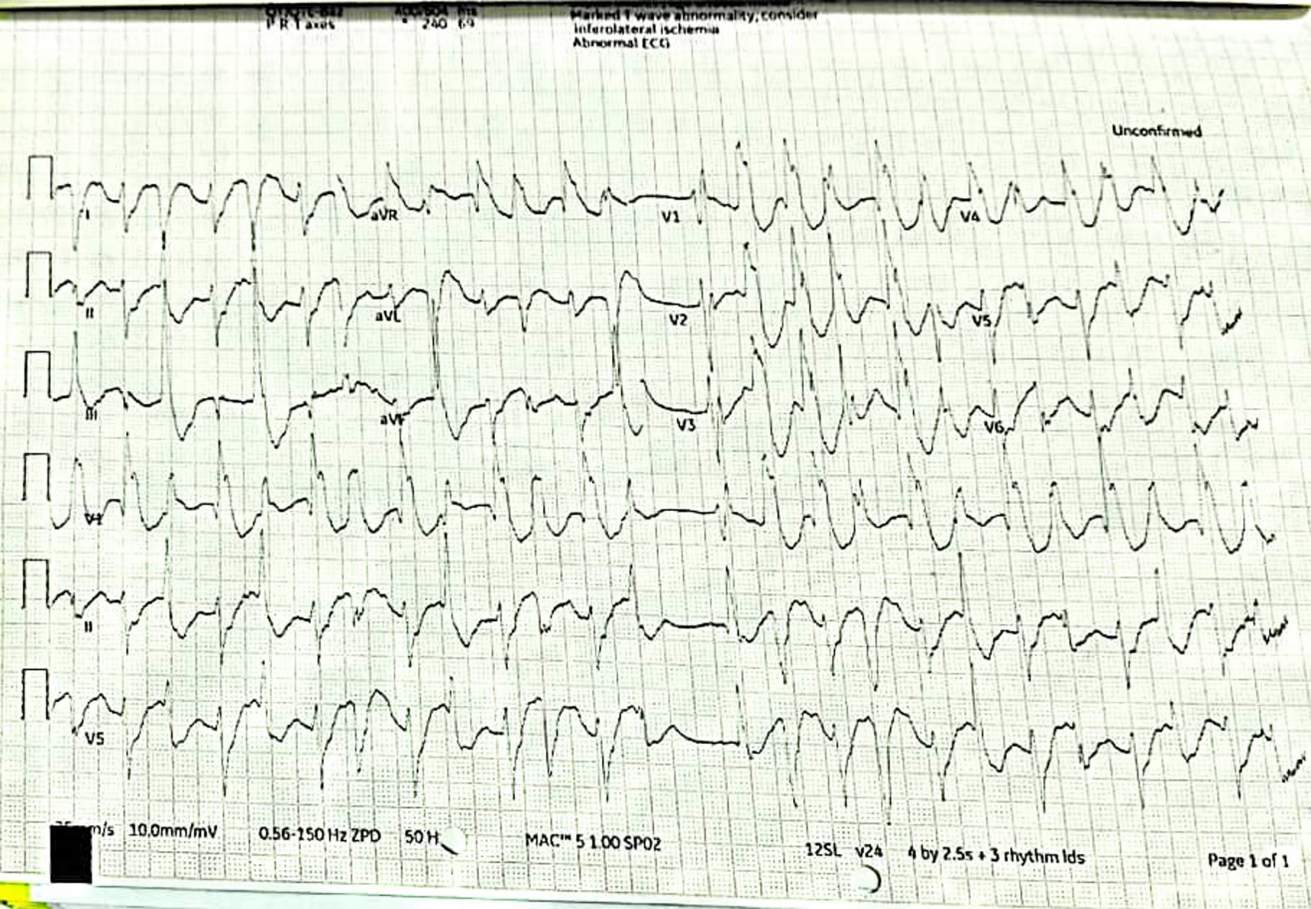
First ECG obtained by emergency doctor at the department.

First electrocardiography (ECG) (Figure 2) revealed a pattern of having multiple multifocal ventricular premature contractions (PCVs). ECG showed ventricular arrhythmia, proceeding to torsades de pointes, ventricular tachycardia and ventricular fibrillation. Based on direct poison ingestion history and ECG evidence, initial management started. In order to adjust the rhythm, a gradual intravenous injection of lignocaine at a dosage of 50 mg was administered. Subsequently, additional doses ranging from 1 to 1.5 mg/kg were given at intervals of 5–10 min, up to a total dosage of 3 mg/kg. This was followed by a continuous infusion at a rate of 1–4 mg/min. This management was carried out for 40 min. Despite these measures, the desired outcome was not immediately achieved. His ECG did not change as a result of it. Based on the clinical presentation of the patient. The consultant recommended to start a treatment with 150 mg IV amiodarone and 1.5 gm magnesium.

**FIGURE 3:**
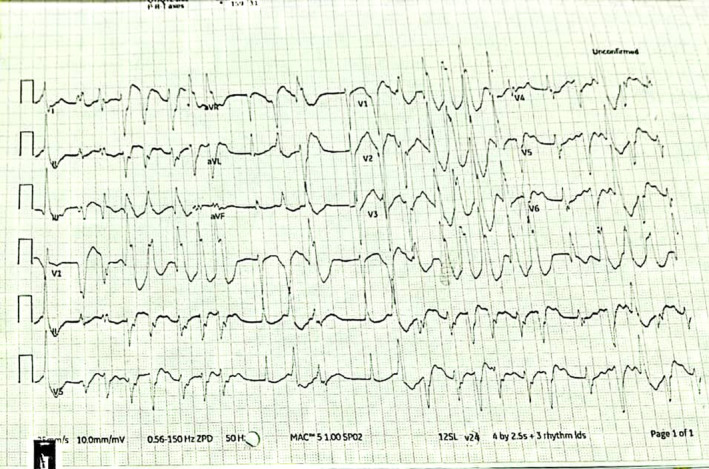
Post‐admission rhythm strips taken at the emergency department.

Following the administration of amiodarone, his blood pressure was 80/60, his pulse rate was 164 bpm, his respiratory rate was 26/min, and his oxygen saturation level was 90%. Premature ventricular contractions (PVCs) were frequently present on a repeat ECG along with atrial fibrillation. (Figure 3).

Throughout the duration, the patient remained unconscious with an unstable blood pressure due to persistent ventricular tachycardia and diminished respiratory effort.

The patient was subsequently taken to intensive care (ICU). While waiting for a bed, the patient experienced a cardiac arrest. Advanced cardiopulmonary resuscitation techniques were commenced, and the initial rhythm recorded by electrocardiography monitoring was ventricular tachycardia, which rapidly proceeded to torsades de pointes and then ventricular fibrillation. At 200 J, synchronized cardioversion was conducted. When 200 J of direct current (DC) shock was administered and cardiopulmonary resuscitation (CPR) was started, the patient's cardiac rhythm became VT. There was ventricular fibrillation. As a result, another 200 J DC shock was administered, and CPR was resumed. Following that, the patient's rhythm revealed ventricular bigeminy.

Twelve minutes later, spontaneous circulation was restored after a total of four defibrillations (200 J), 3 g of magnesium sulfate, and 300 mg of amiodarone were administered. The patient was brought to the intensive care unit with the preliminary diagnosis of Aconite poisoning and was placed on mechanical ventilation.

To preserve cardiovascular stability, he was intubated immediately after rapid sequence intubation (RSI) with ketamine (1 mg/kg), alfentanil (10 mg/kg), and rocuronium (1 mg/kg). Midazolam and fentanyl perfusion were used to maintain anesthesia.

Bilevel‐positive airway pressure (BIPAP) ventilation provided lung‐protective ventilation with tidal volumes no greater than 8 mL/kg and maximal inspiratory pressures no greater than 30 cmH2O. With elevated lactate (4.8 mmol L^−1^) and negative base (−6.9 mmol L^−1^) and a marginally low pH, acid–base balance revealed a marginally low pH.

He remained sedated and received continuing circulatory support via central venous access with exogenous catecholamines for resistant hypotension; noradrenaline (6 mg kg^−1^ min^−1^) in addition to fluid (125 mL min^−1^ Ringers lactate). Within 4 h of being brought to the ICU, the acid–base balance had returned to normal.

Noradrenaline infusion was necessary to keep hemodynamic stability, and continuous changes in electrical rhythm and QRS morphology were noted (bigeminy, frequent ventricular extrasystoles, sinus rhythm, atrial fibrillation, and nodal rhythm). One hundred milligrams of intravenous lidocaine was administered as a bolus, followed by 1.5 g kg min^−1^.

Analyses of cardiac enzymes revealed normal LDH and CK values, as well as troponin levels in the plasma. Normal liver and kidney function was observed. Laboratory measurement of serum electrolytes revealed that potassium (3.7 mmol/L), magnesium (0.77 mmol/L), sodium (142 mmol/L), and calcium (2.22 mmol/L) remained within the normal range. Plasma concentrations of aconite were requested but not examined due to a lack of laboratory resources. Following 12 h of therapy in the intensive care unit, electrocardiography revealed short periods of sinus tachycardia interspersed with atrial fibrillation.

On Day 2 after being admitted to the ICU, he remained intubated and ventilated on BIPAP breathing. After achieving hemodynamic stability (BP 120/65 mmHg, 67 beat.min‐1) the tapering dosages of noradrenaline and amiodarone were discontinued. Three episodes of severe bradycardia lasting less than 1 min were seen in the late evening, and all cleared spontaneously without intervention. The laboratory tests remained normal. He was successfully extubated to a 40% venture mask on Day 3 after being admitted to the ICU; he had a SpO2 of 99% and a respiratory rate of 18 breaths.min‐1 with no brain impairment seen. In typical sinus rhythm, respiratory and cardiovascular parameters were steady. During the evening, there were 3 h of severe agitation with tachypnea, which were controlled with intravenous midazolam. He was examined by the critical care consultant in the early afternoon, and no more level two or three care was necessary.

**FIGURE 4:**
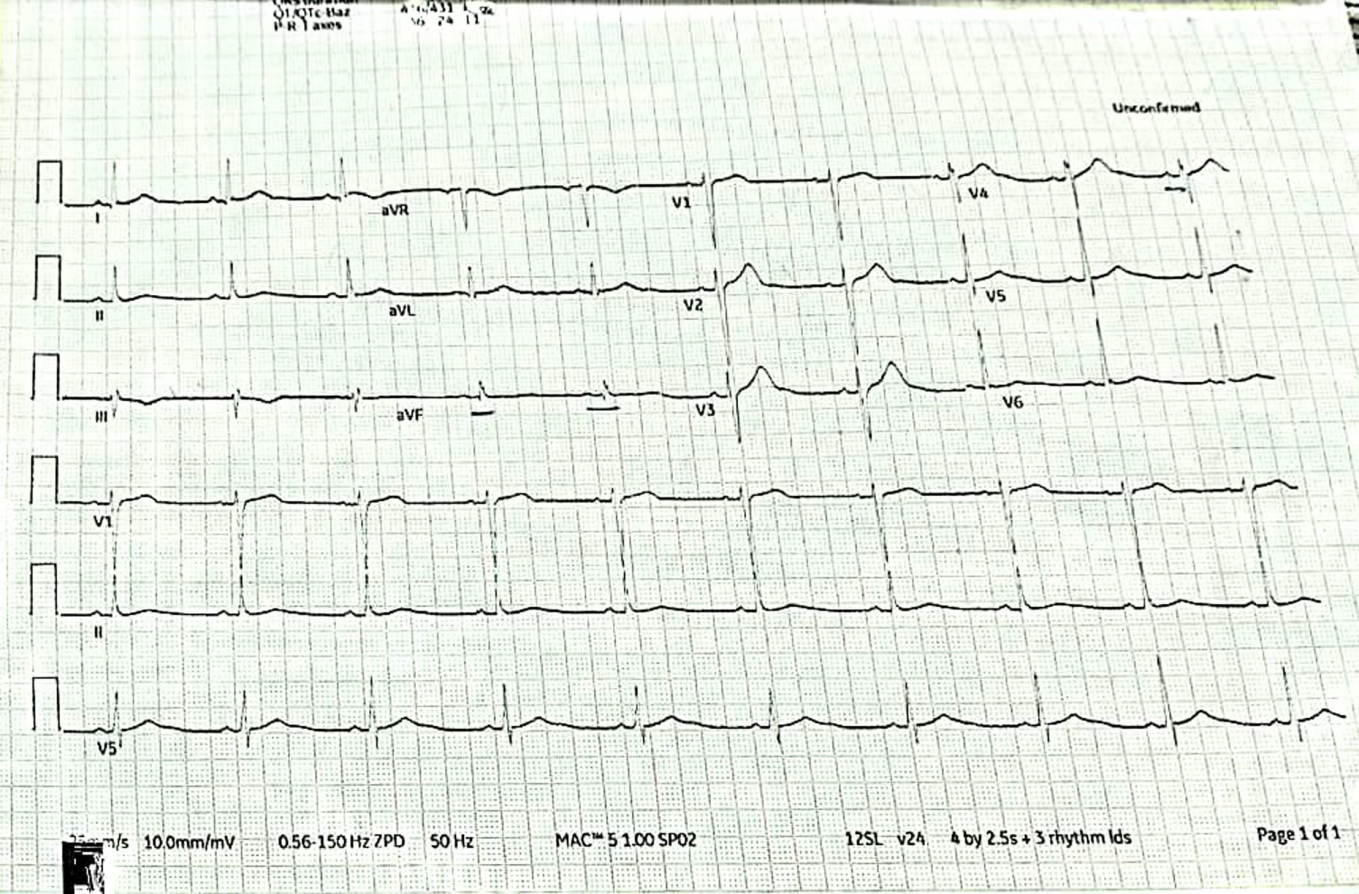
Twelve lead ECG 72 h. Day 3 following admission.

He was sent to a hospital ward for acute care on fourth day and evaluated by psychiatric and general medicine departments. In addition, his ECG had restored to normal sinus rhythm. (Figure 4).

The laboratory tests were repeated on the third and fourth day. His symptoms significantly improved. The patient proceeded to improve and was released from the hospital on the sixth day after admission. Total hospitalization lasted 5 days. The patient was released with 200 mg of oral amiodarone, which was reduced over the course of 2 weeks during following visits. At the time of release, a written patient consent was acquired from the patient. It was conveyed to the patient that his identity would not be disclosed and that the case information will only be utilized for educational purposes. One month later, he reported no shortness of breath, chest discomfort, or palpitations at his follow‐up appointment.

## 3 DISCUSSION

Different complementary and alternative medical practices are being employed in Bangladesh without knowledge of their possible dangers.^8^ However, plant poisoning is a very uncommon condition that may sometimes be fatal. Aconite is frequently found in Asian nations.^5^ Traditional Chinese medicine is an integral component of the healthcare systems in the majority of Asian nations. Aconitum plants and roots are frequently used for their efficacy against musculoskeletal pain, rheumatic diseases, and stomach pain.^9^ It contains a number of alkaloids with strong cardiac and neurotoxic potential. Aconite poisoning is a potentially lethal illness due to its symptoms in overexcited tissues. Aconite, an alkaloid, has a high affinity for voltage‐dependent sodium channels. The poison causes these sodium channels to remain active and depolarized indefinitely, resulting in a number of significant clinical implications.^10^ By overcharging cardiac cells, these pathways can generate severe ventricular arrhythmias such as torsade de pointes or bidirectional ventricular tachycardia.^11^ Case reports and studies have both documented electrocardiographic signs of aconite intoxication.^12^

In our case study, all of these criteria were present, including supraventricular abnormalities with ventricular arrhythmias, multifocal ventricular premature beats, and ventricular tachycardia that swiftly proceeded to torsades de pointes and ultimately ventricular fibrillation upon presentation. Jacobs et al.^13^ reported on a similar presentation with regards to acute collapse and severe cardiovascular instability.

Asian countries have different aconite poisoning causes and preparations (usage of higher‐than‐recommended dosages of 1.5–3.0 g). According to current research, aconite poisonings in Asia were caused by overdose, inadequate processing, usage of aconite tincture, use of crude roots, and lack of standardization in root processing and tincture manufacture.^4^

Toxicity at therapeutic levels is a possibility, and safe dose is determined by processing. The lethal dose for humans is around 5 mg; however, a dose of 2 mg is typically sufficient to cause significant cardiac rhythm problems.^9^ In our case the patient ingested 40 mL of aconite tincture which is about 16 mg of aconite. When antecedent history is unclear, aconite detection in serum might be useful. Various chromatographic procedures are available; however, they are normally unavailable in most laboratories, are time‐consuming, and might result in diagnostic delays.^2^

Because there is no specific antidote, supportive management is the primary therapy for poisoning.^14^ This includes careful vital sign monitoring, supportive cardiovascular treatment such as intravenous hydration, and rhythm management using amiodarone for tachyarrhythmias and atropine for bradycardia.^15^ In addition to supportive care, complications are addressed. Amiodarone, flecainide, procainamide, mexiletine, lidocaine, magnesium sulfate, and other medications, as well as electrical cardioversion, have all been investigated with varying degrees of effectiveness.^16^ Lidocaine is seldom useful in treating Aconitum‐induced ventricular tachyarrhythmias.^17^ Amiodarone and flecainide are first‐line therapies. Because aconite has a half‐life of roughly 3 h, resuscitation with extracorporeal life support is advised if necessary to restore and sustain hemodynamics.

Several successful cases of extracorporeal life support have been recorded.^18^ Coulson et al.^12^ report the treatment of 65 cases of probable aconite poisoning resulting in ventricular dysrhythmias in a recent analysis, concluding that flecainide or amiodarone appeared to be more associated to a return to sinus rhythm than lidocaine and/or cardioversion. Our patient recovered with amiodarone and finally reached normal sinus rhythm, demonstrating the effectiveness of amiodarone in aconite‐induced arrhythmias. This instance underscores the importance of early identification and the use of appropriate anti‐arrhythmic medications, such as amiodarone, to restore normal sinus rhythm. To prevent mortality, cardiac involvement needs early diagnosis and therapy of rhythm abnormalities.

## 4 CONCLUSION

In Asia, aconite alkaloid is commonly used and available herbal medicine. Aconite has a small safety margin between therapeutic and fatal dosages. Although aconite poisoning is rare, it is well‐known for causing fatal cardiac arrhythmias. In acute poisoning, early diagnosis and therapy of heart rhythm disturbances is the mainstay of management. To avoid the negative implications of alternative medicine, educational efforts and ethnic healthcare integration techniques should be considered. Self‐treatment using herbal medications should only be carried out under controlled conditions to prevent adverse reactions.

## AUTHOR CONTRIBUTIONS

**Md. Mahabubul Islam Majumder:** Conceptualization; supervision; writing – review and editing. **Ashrafur Rahaman Mahadi:** Conceptualization; investigation; writing – original draft; writing – review and editing. **Obayed Ur Rahman:** Formal analysis; resources. **Biplob Kumar Roy:** Resources; visualization. **Hossain Mohammad Shihab:** Resources; visualization.

## FUNDING INFORMATION

This research did not receive a particular grant from any governmental, private, or non‐profit funding source.

## CONFLICT OF INTEREST STATEMENT

The authors state that they have no known conflicting financial interests or personal relationships that may be seen as having influenced the work described in this study.

## ETHICS STATEMENT

The Institutional Review Board of Central Medical College in Cumilla, Bangladesh, approved this study. A formal letter was provided as clarification for conducting this case review. For publishing of the clinical details and photographs in this article, signed informed consent from the patient was acquired.

## CONSENT

Written informed consent was obtained from the patient to publish this report in accordance with the journal's patient consent policy.

## Data Availability

All the required information is available in the manuscript itself.
